# SPC Liposomes as Possible Delivery Systems for Improving Bioavailability of the Natural Sesquiterpene β-Caryophyllene: Lamellarity and Drug-Loading as Key Features for a Rational Drug Delivery Design

**DOI:** 10.3390/pharmaceutics10040274

**Published:** 2018-12-13

**Authors:** Antonella Di Sotto, Patrizia Paolicelli, Martina Nardoni, Lorena Abete, Stefania Garzoli, Silvia Di Giacomo, Gabriela Mazzanti, Maria Antonietta Casadei, Stefania Petralito

**Affiliations:** 1 Department of Physiology and Pharmacology “V. Erspamer”, Sapienza University of Rome, P.le Aldo Moro 5, 00185 Rome, Italy; lorena.abete@uniroma1.it (L.A.); silvia.digiacomo@uniroma1.it (S.D.G.); gabriela.mazzanti@uniroma1.it (G.M.); 2 Department of Chemistry and Technology of Drugs, Sapienza University of Rome, P.le Aldo Moro 5, 00185 Rome, Italy; martina.nardoni@uniroma1.it (M.N.); stefania.garzoli@uniroma1.it (S.G.); mariaantonietta.casadei@uniroma1.it (M.A.C.)

**Keywords:** lipophilic compound, caryophyllene sesquiterpene, antiproliferative activity, liposomes, lamellarity, drug loading

## Abstract

The natural sesquiterpene β-caryophyllene (CRY) has been highlighted to possess interesting pharmacological potentials, particularly due to its chemopreventive and analgesic properties. However, the poor solubility of this sesquiterpene in aqueous fluids can hinder its uptake into cells, resulting in inconstant responses of biological systems, thus limiting its application. Therefore, identifying a suitable pharmaceutical form for increasing CRY bioavailability represents an important requirement for exploiting its pharmacological potential. In the present study, the ability of soybean phosphatidylcholine (SPC) liposomes to improve bioavailability and absorption of CRY in cancer cells has been evaluated. Liposomal formulations of CRY, differing for lamellarity (i.e., unilamellar and multilamellar vesicles or ULV and MLV) and for the drug loading (i.e., 1:0.1, 1:0.3 and 1:0.5 mol/mol between SPC and CRY) were designed with the aim of maximizing CRY amount in the liposome bilayer, while avoiding its leakage during storage. The low-loaded formulations significantly potentiated the antiproliferative activity of CRY in both HepG2 and MDA-MB-468 cells, reaching a maximum IC_50_ lowering (from two to five folds) with 1:0.3 and 1:0.1 SPC/CRY MLV. Conversely, increasing liposome drug-loading reduced the ability for CRY release, likely due to a possible interaction between SPC and CRY that affects the membrane properties, as confirmed by physical measures.

## 1. Introduction

**Figure 1:**
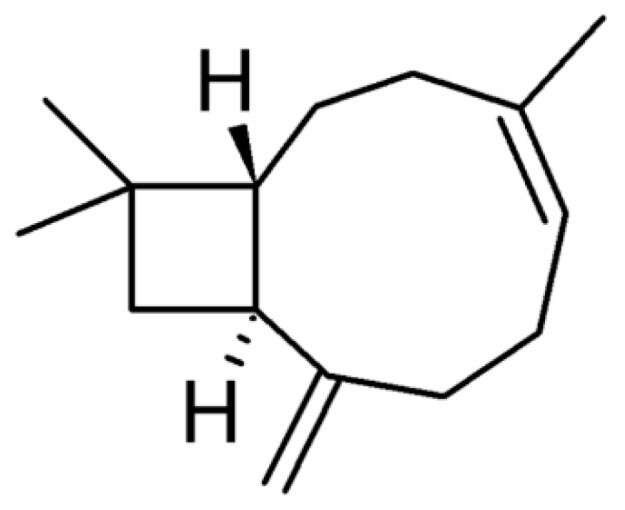
Chemical structure of β-caryophyllene (CRY).

β-caryophyllene or *trans*-caryophyllene (CRY), a bicyclic sesquiterpene with a rare cyclobutane ring (Figure 1), is a volatile compound found in large amounts in the essential oil of many different spice and food plants, particularly *Eugenia caryophyllata* L., *Copaifera multijuga* (copaiba) and *Cannabis sativa* L [1].

In nature, β-caryophyllene is usually found together with small amount of its isomers α-caryophyllene and γ-caryophyllene or in a mixture with its oxidation product, β-caryophyllene oxide. Several biological activities have been reported for β-caryophyllene, including antimicrobial, antileishmanial, antimalarial, local anesthetic, spasmolytic and anticonvulsivant activities [2]. It has been reported to partly act as an agonist of the CB2 receptor, which represents a therapeutic target for the treatment of inflammation, pain, atherosclerosis, and inflammatory-based diseases, including colitis, cerebral ischemia and brain inflammation [3,4,5,6,7]. Also, it has been recently shown to possess chemopreventive properties [1,8,9,10,11] and displayed a chemosensitizing power when administered in combination with anticancer drugs, thus resensitizing chemoresistant cancer cells [12]. It was found to be able to interfere with targeted signalling pathways involved in inflammation and cancer, including HMGB1/TLR4 signalling and STAT3 [10,13,14].

Despite these promising biological activities, β-caryophyllene is characterized by high lipophilicity and poor stability in hydrophilic media (such as biological fluids), which limit its bioavailability and absorption into cells. Bioavailability depends on the nature and chemical-physical properties of a molecule and is mainly due to water solubility (or dissolution rate) and membrane permeability [15]. Low bioavailability is a common feature of different natural substances, defined as “poorly water-soluble drugs”, and can hinder their administration, clinical application and market entry. In this context, improving bioavailability represents an important requirement for exploiting the pharmacological potential of such natural substances and meeting the need for suitable pharmaceutical formulations.

To this end, various strategies, including formulation in complex forms as micelles, liposomes, polymeric nanoparticles and lipid nanoparticles, have been approached. Among them, liposomes have been extensively applied in the years as biomembrane models and as drug carriers in the pharmaceutical and medical fields, owing to their excellent biocompatibility and biodegradability, low toxicity and lack of immunogenicity [16,17]. They have also been adopted as efficient systems for incorporating natural compounds, such as essential oil components, and improving their solubility and chemical stability [18].

Liposome structure allows the incorporation of different types of drugs: hydrophilic substances are encapsulated in the inner aqueous compartments, while lipophilic drugs are mainly entrapped within the lipid bilayer [19]. According to lamellarity and size, they are usually classified as multilamellar vesicles (MLV; greater than 0.5 µm), small unilamellar vesicles (SUV; between 20 and 100 nm) and large unilamellar vesicles (LUV; greater than 100 nm) [20].

Taking into account the strong lipophilicity of CRY and its low dissolution rate in biological fluids, in the present study we propose a rational design of soy phosphatidylcholine (SPC) liposomal formulations for improving cellular uptake of CRY and then its antiproliferative activity in cancer cells, focusing on lamellarity and drug-loading as major key features to develop optimized delivery systems. SPC is commonly used in different types of drug delivery formulations, due to its structural similarity with biomembrane phospholipids, and seems to represent an interesting molecule to be used for designing liposomal chemotherapy formulations, since it could enhance the antiproliferative activity of anticancer drugs by affecting the cholesterol-induced stiffening of cancer cell biomembrane, thus favoring drug permeability. It is well accepted that cancer cells, respect to normal cells, are characterized by changes in biomembrane phospholipid composition and a constitutive activation of the fatty acid biosynthesis seems to support the increased cell proliferation [21]. Particularly, higher accumulation of cholesterol leads to a more rigid and low-permeable membrane, with increased resistance to cancer chemotherapy.

In order to characterize the best features of SPC liposomes in improving the dissolution of CRY in biological fluids and its cellular uptake, both unilamellar (ULV) and multilamellar (MLV) formulations were studied. In fact, due to the physico-chemical properties of CRY, it is expected that the drug is incorporated within the phospholipid bilayer of liposomes. Therefore, liposomal formulations of CRY have been rationally designed taking into account that the loading of CRY in the bilayer of liposomes, while avoiding its leakage during storage, requires special consideration in product development and represents a key feature for optimizing the formulation. The lipid composition, lamellarity, the manufacturing process and drug incorporation can all influence the physicochemical properties of a liposomal formulation, including the drug release performance. Therefore, when liposomes are investigated as drug delivery vehicles of hydrophobic drugs, the influence of the drug–lipid ratio on the arrangement of the nonpolar region of the vesicles membrane should be considered to design a delivery vehicle that is at the same time able to catch and release the encapsulated payload in order to achieve the therapeutic purpose [22].

In line with this evidence, in the present study different formulations at three loading degrees, characterized by SPC phospholipid and CRY molar ratio of 1:0.1, 1:0.3 and 1:0.5 as well as different lamellarity were prepared. A physicochemical characterization by dynamic light scattering, fluorescence anisotropy and entrapment efficiency of CRY were performed. The increased bioavailability was evaluated on the basis of the cytotoxicity potency of the formulations encapsulating CRY with respect to the substance alone. In specific, the antiproliferative activity of CRY-loaded SPC ULV and MLV with respect to that of CRY alone was studied in liver cancer HepG2cells. Also, triple negative MDA-MB-468 breast cancer cells were used being high-responsive to CRY cytotoxicity respect to HepG2 cells.

## 2. Materials and Methods

### 2.1. Chemicals

β-Caryophyllene (CRY; >98.5% purity), soybean phosphatidylcholine (Phospholipon90; SPC), HEPES [4-(2-hydroxyethyl) piperazine-1-ethane-sulfonic acid], thiocyanatoiron (III), 1,6-diphenyl-1,3,5-hexatriene (DPH) and (4,5-dimethyl-2-thiazolyl)-2,5-diphenyl-2H-tetrazolium bromide (MTT; ≥97.5% purity) and cholesterol (Chol) were purchased from Sigma-Aldrich Co (St. Louis, MO, USA). Dulbecco’s Modified Eagle’s medium (DMEM) was from Aurogene (Rome, Italy). Chloroform, dimethyl sulfoxide, ethanol, 1,2-dicloroethane and hydrochloric acid were supplied by Carlo Erba Reagents (Arese, Italy) and were of analytical grade. All solutions were prepared in the better solvent, sterilized by filtration and stored for a just conservation time at recommended temperature, i.e., room temperature (RT) or refrigerated conditions (from 4 °C to −20 °C).

For the cytotoxicity assay, the sesquiterpene was dissolved in absolute EtOH (100% *v*/*v*): at the tested concentrations, and the percentage of ethanol was less than 1% *v*/*v* in the final mixture, in order to exclude a potential toxicity due to the solvent. Conversely, both the CRY-loaded and plain SPC liposomes were directly dispersed in the culture medium at different concentrations.

### 2.2. Liposome Preparation

Liposomes were prepared by the thin-film hydration method followed by extrusion [23]. Specifically, 250 mg of SPC and different amount of CRY (7, 20 or 33 mg) were dissolved in a 50 mL round-bottom flask in the minimum volume of chloroform to give lipid-to-drug molar ratio of 1:0.1, 1:0.3 and 1:0.5.

The solvent was removed by rotary evaporation under reduced pressure to form a thin layer on the flask wall. The resultant thin film was further dried with a high vacuum oil pump for at least 2 h.

Dried film was hydrated in 5 mL of a 10 mM HEPES buffer solution (pH 7.4) at 25 °C and the dispersion was shaken vigorously with a vortex mixer to form multi-lamellar vesicles (MLV).

The generated multilamellar vesicles were repeatedly extruded at 25 °C through polycarbonate membranes of decreasing pore size using a thermobarrel Extruder, (Lipex Biomembrane, Vancouver, BC, Canada) until a defined size distribution was achieved (2 times through 400 nm membranes and finally 6 times through 200 nm membranes). All liposome formulations were flushed with nitrogen gas, stored at 4 °C and used within two weeks.

### 2.3. Gas Chromatographic/Mass Spectrometric (GC/MS)

Purity of CRY and its concentration obtained in the liposomal dispersion was determined by the gas chromatographic/mass spectrometric (GC/MS) technique. The GC/MS analyses were performed on a Clarus 500 series from Perkin Elmer instruments (Waltham, MA, USA) operating in the electron impact mode (70 eV) and equipped with NIST (National Institute of Standards and Technology) libraries. A Stabilwax fused-silica capillary column (Restek, Bellefonte, PA, USA) (60 m × 0.25 mm, 0.25 mm film thickness) was used with helium as carrier gas (1.0 mL/min). 1 µL of sample was injected into the GC injector at the temperature of 280 °C and in splitless mode. The oven of GC was programmed to rise from 90 °C to 200 °C at 3 °C/min and then held at 200 °C for 2 min. All analyses were performed at constant flow. A calibration curve was generated by running various solutions containing graded amounts of the CRY and injecting a constant volume of each standard solution exactly measured. The calibration curve was obtained by plotting the peaks area (automatically calculated by the computer) on the ordinate and the amounts on the abscissa.

### 2.4. Physicochemical Characterization of Liposomes

#### 2.4.1. Dynamic Light Scattering (DLS) and Zeta-Potential Measurements

Particle size distribution and zeta-potential were measured with a Zetasizer Nano ZS90 (Malvern Panalytical, Malvern, UK). Hydrodynamic diameter and polydispersity index were evaluated by dynamic light scattering (DLS) experiments, whereas zeta-potential was measured by electrophoretic light scattering (ELS) experiments. The DLS and ELS techniques used a photon correlator spectrometer equipped with a 4 mW He/Ne laser source operating at 633 nm. All measurements were performed at a scattering angle of 90° and were thermostatically controlled at 25 °C. The samples were opportunely diluted with 10 mM HEPES (pH 7.4) before analysis. Size, polydispersity index and zeta-potential values of the liposome formulations are the mean of three different preparation batches ± standard deviation.

#### 2.4.2. Assay of Phospholipids

Phospholipid content in liposomes was quantified as reported in literature [24]. Briefly, 0.4 mL of sample (20–200 nmol) was mixed with 0.2 mL of ethanol, 1 mL of thiocyanatoiron (III) and 0.6 mL of 0.17 N hydrochloric acid. 3 mL of 1,2-dichloroethane were added to extract the thiocyanatoiron–phospholipid complex formed after shaking for 2 min. The sample was then centrifuged for 5 min at 12,000 rpm. The absorbance of the organic phase was read at a wavelength of 470 nm in a Lambda 25 spectrophotometer (Perkin Elmer, Waltham, USA). The calibration curve was obtained with several solutions of known SPC concentration.

#### 2.4.3. Evaluation of Total Amount of β-Caryophyllene (CRY) in Liposomal Suspensions

Phospholipids and drug molecules dissolved in the organic phase may be get lost during solvent removal under vacuum and high-pressure extrusion steps. Considering that CRY is a liquid with an initial boiling point of 129 °C, to evaluate the amount of the sequiterpene actually present in the different liposomal formulations, it was first extracted from vesicles and then its concentration was determined by the GC/MS technique reported in Section 2.3. In particular, 5 mL of CRY-loaded SPC ULV were diluted with 10 mL of 10 mM HEPES (pH 7.4) and extracted with CHCl_3_ (5 mL). To promote the separation of the two phases, CaCl_2_ (460 mg) was added to the biphasic system and the organic phase then collected. The extraction procedure was repeated three times, all the organic phases were mixed and made up to known volume before GC/MS analysis.

#### 2.4.4. Steady-State and Time-Resolved Fluorescence Measurements

Steady state anisotropy of DPH in liposomes was measured to assess the effect of CRY and its concentration on the fluidity of SPC liposome membranes. To this end, DPH-loaded SPC liposomes were prepared by the thin-film hydration method reported in Section 2.2, dissolving SPC (250 mg) and DPH (0.15 mg; 6.46 × 10^−4^ mmol) in a 50 mL round-bottom flask in the minimum volume of chloroform to give a lipid-to-DPH molar ratio of 1:0.002.

The solvent was removed as previously described and the resultant thin film was hydrated in 5 mL of a 10 mM HEPES buffer solution (pH 7.4) at 25 °C. The generated multilamellar vesicles were repeatedly extruded at 25 °C through polycarbonate membranes of decreasing pore size.

DPH normally is located within the hydrophobic region of the bilayer membrane. DPH responds to changes in physical properties of the acyl chain region of the membrane that affect its ability to rotate. Probe movement is quantified by measuring the degree to which DPH fluorescence emission is depolarized following excitation by polarized light. These fluorescence anisotropy measurements respond to changes in the order degree of the DPH surrounding environment: changes in the liquid–crystalline state organization of the liposome membrane alter the rate of probe movement; in particular, the more disordered the membrane environment, the greater is the motional freedom of the fluorophore and hence the lower the observed anisotropy. An increase in steady state anisotropy of DPH in membranes may imply a reduction in mobility of lipids.

Steady-state fluorescence anisotropy measurements, for DPH-loaded liposomes, were carried out at room temperature with a Perkin-Elmer LS50B spectrofluorometer. The excitation and emission wavelengths were 350 and 450 nm, respectively, and all slits were set to a width of 2.5/2.5 nm.

Samples, opportunely diluted with 10 mM HEPES buffer pH 7.4, were illuminated by vertically (V) or horizontally (H) polarized monochromatic light at λ = 350 nm and the emitted fluorescence intensities (I) parallel or perpendicular to the direction of the excitation beam were recorded at λ = 450 nm. Total fluorescence intensity $\left[I_{f}=\left(I_{⫽}\right)V+2G\left(I_{┴}\right)H\right]$ is obtained by addition of the respectively horizontally $\left[I_{┴}\right]$ and vertically $\left[I_{⫽}\right]$ intensities polarised light emission. The stationary fluorescence anisotropy (r) was determined using the typical calculation:

$$r=\frac{\left(I_{⫽}\right)V-G\left(I_{┴}\right)H}{I_{f}}$$

Total fluorescence intensity and anisotropy measurements required correction for the gain of photomultipler detector $\left[G=\left(I_{⫽}\right)H/\left(I_{┴}\right)H\right]$.

The effect of CRY incorporation on the phospholipid bilayer of SPC liposomes was evaluated by comparison with the well-known effect produced by cholesterol (Chol) on the membrane behavior.

Anisotropy data are represented as the mean ± standard deviation (SD).

### 2.5. Cytotoxicity Studies

#### 2.5.1. Human Cancer Cell Lines

Liver cancer HepG2 cells were a kind gift of Prof. Eufemi (Sapienza University of Rome, Italy), while triple negative MDA-MB-468 breast cancer cells were purchased from IRCCS AOU San Martino -IST (Genoa, Italy). The cells were grown under standard conditions (37 °C and 5% CO_2_) in DMEM-F12 medium containing L-glutamine (1% *v*/*v*) and HEPES (15 mM) and supplemented with 10% heat-inactivated FBS, 100 U/mL penicillin and 100 μg/mL streptomycin in 75 cm^2^ flasks. Subcultures were prepared every 4 days, renewing growth medium every 2–3 days. All experiments were performed when cells reached the logarithmic growth phase.

#### 2.5.2. Cytotoxicity Assay

The cultured cells were seeded into 96-well microplates (20,000 cells/well), allowed to grow for 24 h, then treated with CRY (1–75 µg/mL in EtOH 1% *v*/*v*) or CRY loaded SPC liposomes. The concentrations of pure CRY were prepared by progressive dilution in EtOH 100% *v*/*v*, then added to cells at 1% *v*/*v* in the final mixture, at which ethanol was nontoxic. A vehicle control (EtOH 1% *v*/*v* in the final mixture for CRY and 10 mM HEPES for SPC ULV and MLV), corresponding to 100% cell viability and a standard cytotoxic agent (i.e., doxorubicin, 10 µg/mL in the final mixture) were also included in the experiments.

After 24 h incubation, the cytotoxicity of the treatment was measured by the 3-[4,5-dimethylthiazol-2-yl]-2,5-diphenyl tetrazolium bromide (MTT) assay according to previous published methods [25]. The assay was carried out at least in three biological replicates, and in each experiment each concentration was tested in six technical triplicates. The treatment was considered cytotoxic when the cell viability was lower than 70% with respect to vehicle treated cells [26].

The results were expressed as percentage of cell viability (about three experiments including 8-10 replicates for each treatment) with respect to the vehicle.

Results of cytotoxicity studies are expressed as mean ± standard error (SE). The concentration–response curves were constructed using the Hill equation:

$$E=\frac{E_{max}}{\left[1+\left(10logIC_{50}-A\right)Hill\;Slope\right]}$$

where *E* is the effect at a given concentration of the substance, *E_max_* is the maximum activity, *IC*_50_ is the concentration that produces a 50% of the inhibitory response, *A* is the substance molar concentration, *HillSlope* is the curve slope.

### 2.6. Statistical Analysis

Statistical analysis was performed by GraphPad Prism™ (Version 4.00) software (GraphPad Software, Inc., San Diego, CA, USA). The one-way analysis of variance (one-way ANOVA), followed by Dunnett’s multiple comparison post-test, was used to analyze the difference among different treatments, while the Student’s *t*-test was applied to determine the statistical significance between two different experimental conditions. The values of *p* < 0.05 were considered significant.

## 3. Results

### 3.1. Physicochemical Characterization of Soybean Phosphatidylcholine (SPC) Unilamellar and Multilamellar Vesicles (ULV and MLV)

Results obtained by DLS measurements highlighted that high pure CRY did not alter the vesicles formation, since no significant changes in physicochemical features (i.e., mean diameter, zeta-potential and size distribution) of CRY-loaded SPC ULV and MLV, compared to conventional liposomes, were found even at the highest molar ratio tested (1:0.5 mol/mol) (Table 1 and Table 2). Moreover, no loss of CRY was observed during the preparation process. In fact, GC/MS analysis performed after the extraction of CRY from liposomes evidenced a perfect overlap of the actual recovered amount with the theoretical one, thus indicating that no losses of sesquiterpene occurred. When the physicochemical features of liposomes were evaluated in the presence of cell medium, no interference with the dimensional analysis was highlighted, thus suggesting a suitable stability of the formulation in cell culture environment (data not shown). The amount of structured phospholipids in the liposomal suspensions, with and without CRY, resulted in not being affected by the presence of the sesquiterpene, thus suggesting their compatibility.

### 3.2. Cytotoxicity of CRY and Plain SPC-Based Liposomes

**Figure 2:**
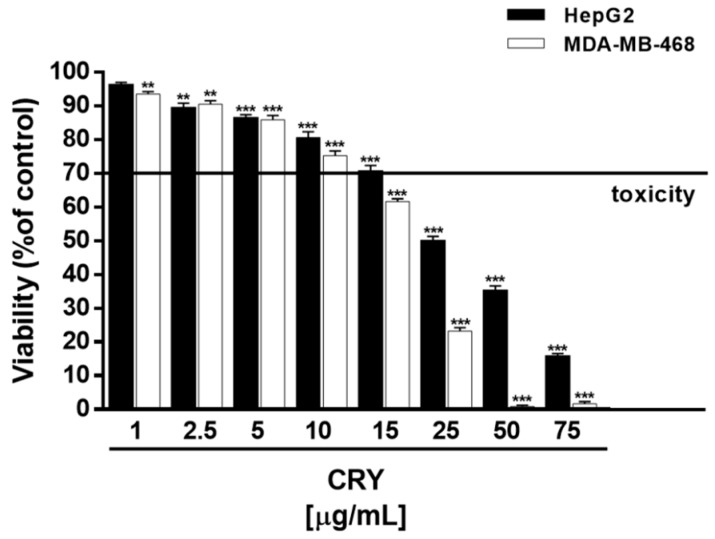
Effect of CRY on the viability of HepG2 and MDA-MB-468 cells. ** *p* < 0.01 and *** *p* < 0.001 (analysis of variance (ANOVA) + multiple Dunnett’s comparison post-test); denotes a statistically significant reduction of cell viability compared to control (i.e., vehicle-treated cells). A cell viability lower than 70% respect to control was considered as cytotoxic [26].

Under our experimental conditions, CRY did not affect the cell viability of HepG2 cells up to 10 μg/mL, although a slight cell viability reduction (about 10%) was found starting from 5 μg/mL. A significant decrease of cell viability (about 45% reduction respect to control) was found at 25 μg/mL, reaching a greatest inhibition of about 90% at 75 μg/mL (Figure 2). In MDA-MB-468 cells, CRY produced early toxicity signs (inhibition of 10%) at 2.5 μg/mL, with a biologically significant effect at 15, 25 and 50 μg/mL (inhibition of 38%, 84% and 98% respectively) (Figure 2). The IC_50_ values were 44.7 (C.L. 19.5–96.8) and 19.2 (C. L. 15.4–23.8) μg/mL in HepG2 and MDA-MB-468 cells, respectively.

**Figure 3:**
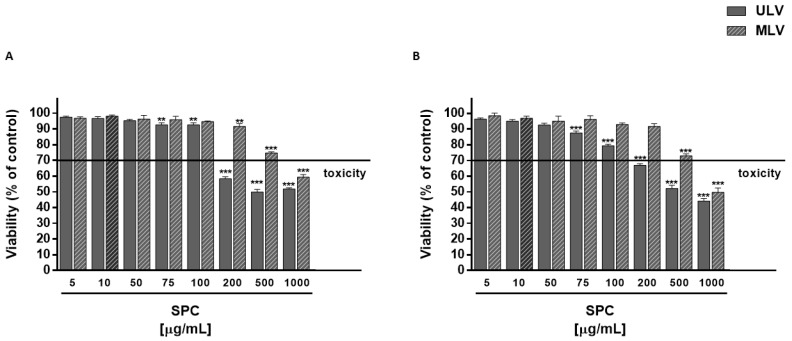
Effect of SPC ULV and MLV on the viability of HepG2 (**A**) and MDA-MB-468 (**B**) cells. ** *p* < 0.01 and *** *p* < 0.001 vs. control (i.e., vehicle-treated cells), denotes a statistically significant reduction of cell viability compared to control (ANOVA + multiple Dunnett’s comparison post-test). A cell viability lower than 70% respect to control was considered as cytotoxic [26].

The cytotoxicity of plain SPC ULV and MLV (1–1000 μg/mL) in HepG2 and MDA-MB-468 cells was preliminarily evaluated, in order to define the maximum concentration at which liposomal formulations did not affect cell viability. Plain SPC ULV were nontoxic up to the concentration of 100 μg/mL in both HepG2 and MDA-MB-468 cells, with biologically significant cytotoxic effects (from 40% to 56% inhibition of cell viability) starting from 200 μg/mL (Figure 3). Conversely, plain SPC MLV produced biologically significant cytotoxic effects (from 40% to 50% inhibition of cell viability) at 1000 μg/mL in both HepG2 and MDA-MB-468 cells, with early toxicity signs (about 25% inhibition of cell viability) at 500 μg/mL (Figure 3).

### 3.3. Cytotoxicity of CRY-Loaded SPC ULV at Different Molar Ratio

**Figure 4:**
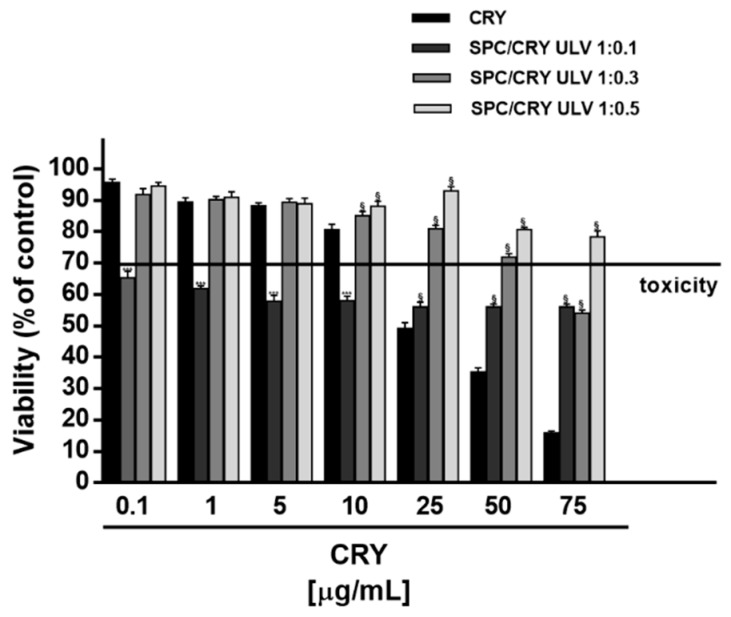
Cytotoxicity of SPC ULV differently loaded with CRY (1:0.1, 1:0.3 and 1:0.5 mol/mol) in HepG2 cells after 24 h incubation. *** *p* < 0.001 vs CRY; denotes a statistically significant increase of cytotoxicity respect to pure CRY (ANOVA + multiple Dunnett’s comparison post-test). ^§^ *p* < 0.001 vs. CRY, denotes a statistically significant reduction respect to CRY cytotoxicity (ANOVA + multiple Dunnett’s comparison post-test). A cell viability lower than 70% respect to control (i.e., vehicle-treated cells) was considered as cytotoxic [26].

When assessed in HepG2 cells, different behaviour was found for CRY-loaded SPC ULV respect to pure CRY, as a function of their molar ratio (Figure 4). In particular, the 1:0.1 molar ratio between SPC and CRY produced about a 40% cytotoxicity increase of CRY at low concentrations of 0.1, 1, 5 and 10 μg/mL, which were non-effective when CRY was administered as pure compound. Conversely, a progressive loss of the cytotoxic effect of CRY was found at highest concentrations of 25, 50 and 75 μg/mL of 1:0.1 SPC/CRY ULV, reaching a maximum 40% inhibition (Figure 4). In spite of a biologically significant cytotoxicity of low-dose 1:0.1 SPC/CRY ULV, the 1:0.3 and 1:0.5 unilamellar formulations produced non-biologically relevant cytotoxic effects and progressively reduced the antiproliferative activity of pure CRY at all the tested concentrations, reaching a maximum of 63% inhibition (Figure 4).

**Figure 5:**
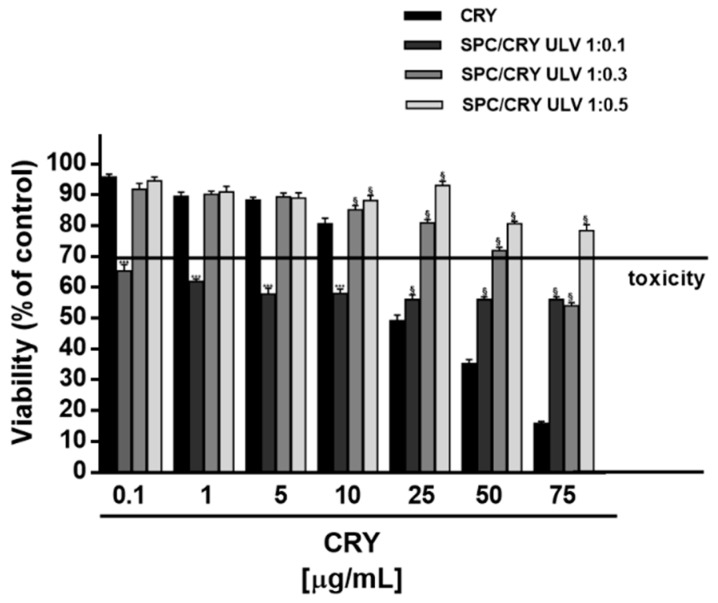
Cytotoxicity of SPC ULV differently loaded with CRY (1:0.1, 1:0.3 and 1:0.5 mol/mol) in MDA-MB-468 cells after 24 h incubation. *** *p* < 0.001 vs. CRY, denotes a statistically significant increase of cytotoxicity respect to pure CRY (ANOVA + multiple Dunnett’s comparison post-test). ^§^ *p* < 0.001 vs. CRY, denotes a statistically significant reduction respect to CRY cytotoxicity (ANOVA + multiple Dunnett’s comparison post-test). A cell viability lower than 70% respect to control (i.e., vehicle-treated cells) was considered as cytotoxic [26].

Furthermore, the highest doses of loaded liposomes (starting from 25 μg/mL of 1:0.1 and 1:0.3 SPC/CRY ULV) significantly reduced the cytotoxicity of plain SPC ULV, at the corresponding concentrations (Figure 4). This evidence suggested that increasing molar ratio between SPC and CRY in ULV formulations can hinder CRY release and retain the molecule into liposomes; also, some interactions between SPC and CRY could be expected, so explaining the reduced toxicity of plain SPC ULV. A similar behaviour was observed in MDA-MB-468 cells for 1:0.5 SPC/CRY ULV, which did not potentiate CRY cytotoxicity in all the experimental conditions, while inhibiting the antiproliferative activity of pure CRY by about 20%, at concentrations of 25 and 50 μg/mL (Figure 5). Conversely, the 1:0.1 loaded formulation induced a significant potentiation of CRY cytotoxicity (from about 20 to 30%) at concentrations of 2.5 and 5 μg/mL, which disappeared at the highest tested concentrations.

Similarly, the 1:0.3 SPC/CRY ULV produced a slight (about 10%) but significant increase of CRY toxicity at concentrations of 2.5 and 5 μg/mL. It is noteworthy that potentiation occurred at non-effective concentrations of CRY administered as a pure compound. For all loaded formulations, the highest concentrations of 25 and 50 μg/mL induced a significant inhibition (from about 10 to 28%) of CRY antiproliferative activity, thus suggesting a possible loss of activity of the drug or the loss of the carrier’s ability to deliver the incorporated substance (Figure 5). When CRY was administered as 1:0.1 SPC ULV, the IC_50_ values were not evaluable in HepG2 cells, while a slight reduction (about 1.5 folds) was obtained in MDA-MB-468 cells (Table 3).

### 3.4. Cytotoxicity of CRY-Loaded SPC MLV at Different Molar Ratio

**Figure 6:**
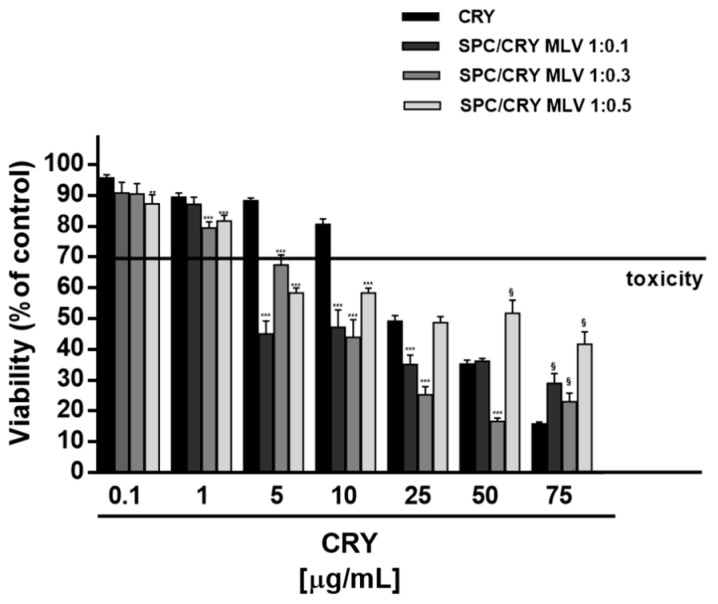
Cytotoxicity of SPC MLV differently loaded with CRY (1:0.1, 1:0.3 and 1:0.5 mol/mol) in HepG2 cells after 24 h incubation. ** *p* < 0.01 and *** *p* < 0.001 vs CRY; denotes a statistically significant increase of cytotoxicity respect to pure CRY (ANOVA + multiple Dunnett’s comparison post-test). ^§^ *p* < 0.001 vs. CRY, denotes a statistically significant reduction respect to CRY cytotoxicity (ANOVA + multiple Dunnett’s comparison post-test). A cell viability lower than 70% respect to control (i.e., vehicle-treated cells) was considered as cytotoxic [26].

Under our experimental conditions, multilamellar liposomes produced different cytotoxic effects as a function of drug loading. In HepG2 cells, 1:0.1 SPC/CRY MLV did not affect the cell viability up to 1 μg/mL, while it produced a marked potentiation of CRY cytotoxicity, at concentrations from 5 to 25 μg/mL, reaching the greatest effect of about 45% at 5 μg/mL (Figure 6). A significant and progressive increase of CRY antiproliferative activity, with a maximum potentiation of about 40%, was also produced by 1:0.3 loaded multilamellar formulation (Figure 6) within the concentrations of 1 and 50 μg/mL: the IC_50_ value of CRY was reduced about five-fold (Table 3). Conversely, the 1:0.5 molar ratio between SPC and CRY increased the biological activity of CRY at concentrations of 5 and 10 μg/mL, with a maximum potentiation of 30% at 5 μg/mL (Figure 6). At the highest concentration of 75 μg/mL, all the CRY-loaded multilamellar formulations markedly reduced the antiproliferative activity of pure CRY, reaching a maximum 26% inhibition (Figure 6).

**Figure 7:**
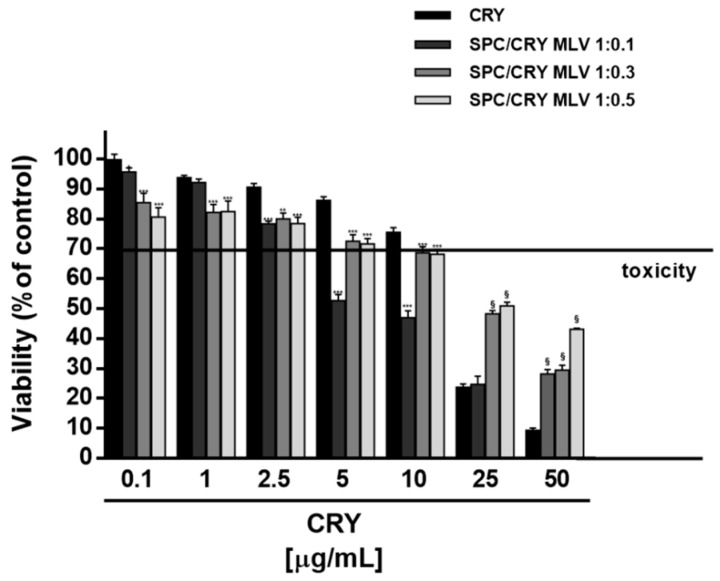
Cytotoxicity of SPC MLV differently loaded with CRY (1:0.1, 1:0.3 and 1:0.5 mol/mol) in MDA-MB-468 cells after 24 h incubation. ** *p* < 0.01 and *** *p* < 0.001 vs. CRY, denotes a statistically significant increase of cytotoxicity respect to pure CRY (ANOVA + multiple Dunnett’s comparison post-test). ^§^ *p* < 0.001 vs. CRY, denotes a statistically significant reduction respect to CRY cytotoxicity (ANOVA + multiple Dunnett’s comparison post-test). A cell viability lower than 70% respect to control (i.e., vehicle-treated cells) was considered as cytotoxic [26].

Analogously, in MDA-MB-468 cells, the 1:0.1 SPC/CRY MLV induced a significant potentiation of CRY cytotoxicity from 0.1 to 10 μg/mL, with a maximum increase of about 30% at 5 and 10 μg/mL (Figure 7). The 1:0.3 and 1:0.5 loaded formulations produced lower potentiation of CRY activity (about 10–25%) from 0.1 to 10 μg/mL. Conversely, the highest concentrations of 25 and 50 μg/mL reduced the antiproliferative activity of pure CRY, reaching a maximum 30% inhibition (Figure 7).

Within the concentrations from 0.1 to 25 μg/mL, the IC_50_ value of CRY was reduced about four-fold when CRY was administered as 1:0.1 MLV, while about two-fold when CRY was administered as 1:0.3 MLV (Table 3).

Furthermore, the highest dose (75 μg/mL) of 1:0.5 SPC/CRY MLV significantly reduced the cytotoxicity of plain SPC MLV at the corresponding concentration, thus displaying a behaviour similar to that found for CRY-loaded ULV (Figure 7). Accordingly, a possible segregation of CRY into liposome due to its interaction with SPC could be expected.

### 3.5. Evaluation of the Potential Interaction between CRY and SPC ULV

In order to better characterize the possible interaction between CRY and SPC ULV, additional experiments in which the two separate components were co-administered to cells, at the same concentrations found in liposome formulations, were performed in HepG2 cells.

**Figure 8:**
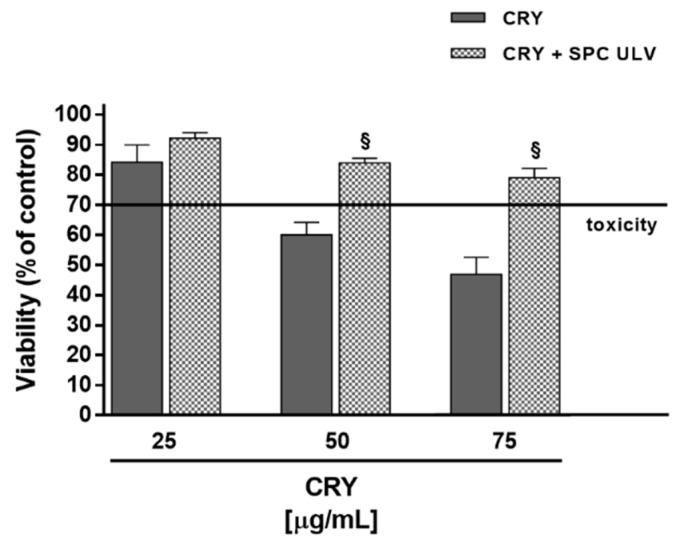
Cytotoxicity of CRY and the combination of SPC/CRY ULV (1:0.5 mol/mol) in HepG2 cells after 24 h incubation. ^§^ *p* < 0.001 vs. CRY, denotes a statistically significant reduction respect to CRY cytotoxicity (ANOVA + multiple Dunnett’s comparison post-test). A cell viability lower than 70% respect to control (i.e., vehicle-treated cells) was considered as cytotoxic [26].

When administered in the presence of SPC ULV, CRY exhibited a lower cytotoxic behaviour with respect to the pure compound. Despite a biologically relevant toxicity of the pure compound alone, the presence of SPC ULV induced a significant loss of CRY bioactivity. About a 30% reduction of its antiproliferative effect is observed (Figure 8) probably due to the high hydrophobic nature of CRY, which is therefore carried off by liposomes.

Similar results were reported by Botré et al. [27], who observed that the addition of empty liposomes to urine samples containing free steroids interfere with the recovery of the drugs, with consequent important implication on doping analysis. These results support our hypothesis that the reduced bioactivity of CRY, when administered as SPC ULV with low molar ratio or at high concentration of the drug, could be due to a close interaction of the substance with phospholipid components as a consequence of its high-grade lipophilicity.

**Figure 9:**
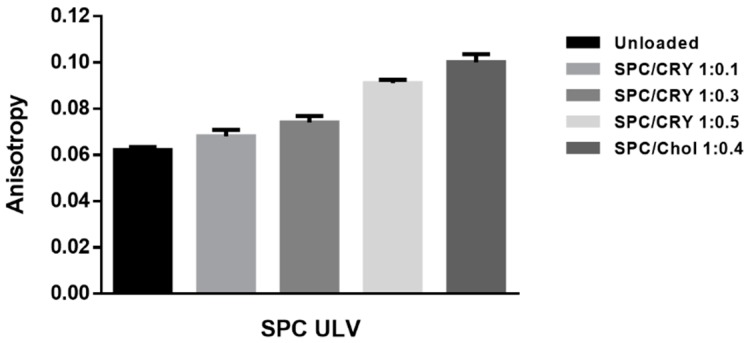
Anisotropy measures obtained by DPH-probe fluorescence for 1:0.1, 1:0.3 and 1:0.5 SPC/CRY ULV and 1:0.4 SPC/cholesterol (Chol) ULV.

To test this hypothesis, fluorescence anisotropy studies were carried out in order to evaluate the effect of CRY and its concentration on the fluidity of the SPC bilayer membrane of liposomes. The results obtained were compared with that produced by cholesterol (Chol) on the membrane behavior (Figure 9). Fluorescence anisotropy studies have found that lipophilic molecules, such as cholesterol and caryophyllene sesquiterpene, can affect the typical fluidity of SPC-based vesicles and reduce the mobility of the hydrocarbon chains of membrane fatty acids. In order to determine how CRY affects membrane structure, we analyze the extent to which this molecule mimics the behavior of cholesterol in *L*_d_ liposomes. Liposomes made of SPC, SPC/Chol and SPC/CRY respectively were tested.

As shown in Figure 9, conventional SPC ULV liposomes produced an average anisotropy value of 0.062. SPC bilayer was expected to be in a homogenous liquid disordered (*L*_d_) state; the low anisotropy value obtained reflects the freedom of movement of the DPH fluorophore in the fluid disordered state of plain SPC liposome.

The thickening effect obtained as a result of cholesterol addition to a fluid bilayer is well documented [27] and the results reported in Figure 9 are in good agreement with previous published data. When cholesterol 40% mol/mol was incorporated in *L*_d_ SPC/Chol bilayer, in fact, the DPH fluorescence anisotropy increases, thus indicating that at this cholesterol amount, a greater order level exists for the phospholipid acyl chain packing. According to Marsh [28], cholesterol in the liposomal fluid bilayer promotes the formation of a phase that coexists with the *L*_d_ phase. In SPC/Chol liposomes the close contact between the sterol and adjacent phospholipids results in the formation of the so called liquid-ordered (*L*_o_) phase. This new intermediate fluid phase exhibits translational degrees of freedom of the lipid molecules that are similar to those in a conventional fluid bilayer state, while the conformational degrees of freedom of the lipid hydrocarbon chains resemble those of the gel state. In contrast, a progressive increase of the anisotropy value appeared for CRY-loaded liposomes that is proportional to the amount of drug incorporated into the vesicles bilayer.

Increasing the mixing ratios between SPC and CRY, the fluorescence anisotropies of DPH were increased almost linearly, suggesting that rotational motion of DPH in SPC liposomes was restricted by CRY entrapment. Comparing the behaviour of SPC/CRY ULV with a 1:0.5 molar ratio and that of SPC/Chol ULV, very similar anisotropy values were found (Figure 9).

In line with these results, our hypothesis is that liposome formulations with a higher than 1:0.1 molar ratio between SPC and CRY can modify the phospholipid bilayer organization, thus hindering drug release.

## 4. Discussion

Low solubility of the natural sesquiterpene β-caryophyllene (CRY) in aqueous fluids and the subsequent poor bioavailability represent key aspects limiting its use in therapy. CRY also exhibited sensitivity to light, oxygen, humidity, and high temperatures [29]: these conditions decrease its stability and limit its biological effectiveness. A possible strategy to overcome these problems is the use of drug delivery systems, which may provide much higher bioavailability of this compound and ensure obtaining desired biological effects. Previous studies proposed cyclodextrin complexation to improve the bioavailability of CRY [30,31,32]. Recently, oil/water microemulsions have been also reported to possess suitable properties for effective topical delivery of β-caryophyllene [33].

Many synthetic and herbal drugs possess the problem of poor oral bioavailability, due to their very low water solubility or poor permeation through the biological membranes, thus leading to a limited dissolution profile in biological fluids and inefficacy in therapy. Increased reports highlighted the promising role of phospholipid-based formulations as effective drug delivery systems for natural bioactive constituents [34]. Being the main components of cellular membrane, phospholipids are characterized by an excellent biocompatibility; also, they possess amphiphilic structures and surface-active wetting characteristics, which allow enhancing the hydrophilicity of hydrophobic compounds. In water, phospholipids self-assemble into supramolecular aggregates, among which liposomes displayed high cell affinity and tissue compatibility, improving drug stability and ability to deliver both hydrophilic and lipophilic substances [34].

In line with this evidence, in the present study the internalization of CRY into the cells was increased using soybean phosphatidylcholine (SPC) liposomes Our hypothesis was that CRY, when administered as liposomal formulations, rationally designed in term of drug to lipid ratio, can be easily uptaken from cells, thus leading to an improved antiproliferative activity. SPC has been used as phospholipid molecule forming the lipid bilayer not only for its structural similarity with cell biomembrane constituents, but also for its ability to affect the cholesterol-induced stiffening of cancer cell biomembrane, which has been found responsible for reduced drug permeability and chemoresistance development in cancer cells [21].

In our cancer cell models, high concentrations of SPC exhibited early cytotoxicity signs, likely due to its ability to increase the permeability of cancer cell biomembrane, through the interference with cholesterol accumulation. In line with this evidence and considering that previous studies highlighted the ability of phosphatidylcholine to induce cholesterol depletion and to be inversely related to cholesterol amount [35,36], we hypothesize that the cytotoxicity found at higher concentrations of plain SPC liposomes can be, at least in part, due to a cholesterol transfer between liposomes and cells. Liposomes could reduce the cholesterol/phospholipid ratio and increase lipid disorder in cell membrane. Depletion of cholesterol in cell thus, could make cell more vulnerable, also to physical stress [37]; similarly, methyl-beta-cyclodextrin has been used for cholesterol removal, although the effect was incomplete [38].

Under our experimental conditions, the encapsulation of CRY within SPC liposomes highlighted that the cellular uptake of CRY can be improved or reduced as a function of the molar ratio between SPC and CRY.

Characterizing the optimal concentration of CRY in liposomal formulations represents a key point for the increase of its antiproliferative effectiveness. Both ULV and MLV liposomes loaded with the highest molar ratio between SPC and CRY (i.e., 1:0.5) induced a progressive loss of the antiproliferative potential of the sesquiterpene. The results obtained highlighted that, despite a possible improvement of low-dose CRY cytotoxicity, increased molar ratio between SPC and CRY markedly interfere with the biological activity of pure compound. This phenomenon suggests that the substance can be greatly retained and become stuck into liposomes, likely due to a stable interaction between CRY and phosphatidylcholine and a stiffening of the membrane structure, which hinders the sesquiterpene release by vesicles and its uptake into cells. Accordingly, Sarpietro et al. [39] showed that CRY possessed a great capacity to interact with membrane phospholipids and to spread through the lipophilic matrix, determining an alteration of the cooperativity. This hypothesis is also in agreement with the more accepted ordering effect induced by cholesterol in biomembrane bilayer and SPC ULV [28]. Therefore, we hypothesize that incorporating high concentrations of CRY in the ULV bilayer can alter membrane packing of SPC liposomes by inducing conformational phospholipid ordering, thus leading to a decreased fluidity and permeability of the bilayer of the phospholipid carrier.

The enhanced rigidity of the membrane, that could be generated by the inclusion of hydrophobic CRY, might seal the system and reduce the drug release. Moreover, the release of high CRY concentrations embedded within the lipid bilayers could be compromised due to strong hydrophobic interactions that might develop between the drug and the phospholipid acyl chains.

The trend displayed by SPC multilamellar vesicles support the hypothesis of the reduced bioactivity of CRY due to a condensing effect of this molecule on the bilayer. In fact, the results observed with the MLV system were partly similar to SPC ULV only at the highest CRY concentrations. We reasonable speculate that CRY reduces the fluidity of the SPC MLV bilayer to a lesser extent with respect to SPC ULV. In fact, for a fixed SPC/CRY ratio, the number of CRY molecules included in each of the lamella of the multilamellar carrier was smaller with respect to ULV, thus leading to a reduced extent of the stiffness effect.

## 5. Conclusions

In the present study, SPC liposomes have been developed as potential effective delivery systems to increase the in vitro bioavailability and stability of the natural sesquiterpene CRY. According to widely accepted evidence that release is governed by molecule lipophilicity and liposome features, our results allow us to hypothesize that CRY release by SPC liposomes is strictly dependent on lamellarity and drug-to-lipid ratio. Lipid-to-drug ratio is a critical parameter as it may influence the therapeutic efficacy of the drug, in particular with lipophilic drugs. The increase of drug encapsulation could be not a valid strategy in liposomal formulation, as the drug release properties of the liposomal product could be negatively affected by the molar concentration of drug in liposomes. In fact, as SPC liposome loading increases, a condensing effect of the loaded molecule on the fluid bilayer occurs. As a consequence, the substance can be greatly retained, and its release restrained. In the case of CRY, this feature can negatively affect and hinder its anticancer effectiveness. Therefore, the SPC to CRY ratio seems to represent a key tool for the development of optimized liposomal formulation to control membrane fluidity and permeability of SPC liposomes, and thus predict the release abilities. In fact, low-loaded MLV appears to induce the maximum increase of CRY antiproliferative activity in both HepG2 and MDA-MB-468 cells, thus suggesting the ability of these formulations to better carry the lipophilic cargo inside cells. However, further in vitro studies are necessary to measure the CRY release by SPC vesicles and the achieved intracellular levels effectively.

In conclusion, our results highlight the importance of rationally designing formulations to develop the optimal liposomal composition, taking into account not only the chemical nature of the payload but also that the high drug loading in liposomes could be critical for the maximum usefulness of its therapeutic potential.

## Figures and Tables

**Table 1:** Physicochemical features of soybean phosphatidylcholine (SPC) unilamellar vesicles (ULV) liposomes.

Sample	Hydrodynamic Diameter (nm)	PdI	ζ-Potential (mV)	SPC Recovery (%)
Unloaded SPC vesicles	180.6 ± 4.7	0.079 ± 0.015	−15.3 ± 0.4	92.2 ± 1.7
SPC/CRY vesicles (mol/mol)				
1:0.1	185.5 ± 4.2	0.076 ± 0.006	−14.1 ± 0.2	89.5 ± 1.2
1:0.3	176.7 ± 7.5	0.075 ± 0.005	−14.3 ± 0.5	88.5 ± 0.5
1:0.5	181.4 ± 2.1	0.085 ± 0.012	−13.5 ± 0.6	85.2 ± 2.2

Hydrodynamic diameter (Z-Average, nm); polydispersity index (PdI); ζ-potential (mV) and % of SPC recovery.

**Table 2:** Physicochemical features of SPC multilamellar vesicles (MLV) liposomes.

Sample	Hydrodynamic Diameter (nm)	PdI
Unloaded SPC vesicles	699.7 ± 6.6	0.359 ± 0.069
SPC/CRY vesicles (mol/mol)		
1:0.1	643.9 ± 13.6	0.339 ± 0.045
1:0.3	631.5 ± 21.1	0.371 ± 0.005
1:0.5	497.1 ± 7.9	0.387 ± 0.025

Hydrodynamic diameter (Z-Average, nm); polydispersity index (PdI).

**Table 3:** IC_50_ values of CRY administered as pure compound or as ULV and MLV formulations in HepG2 and MDA-MB-468 cells.

	HepG2	MDA-MB-468
	IC_50_ (CL) μg/mL *RR*	
	
CRY	44.7 (19.5–96.8)	19.2 (15.4–23.8)
SPC/CRY ULV (mol/mol)		
1:0.1	ne	12.4 (6.4–109.9) *1.5*
1:0.3	ne	ne
1:0.5	ne	ne
SPC/CRY MLV (mol/mol)		
1:0.1	ne	4.9 (2.4–9.9) *3.9*
1:0.3	9.1 (2.6–32.0) *4.9*	8.3 (6.1–11.2) *2.3*
1:0.5	ne	ne

CL, confidential limits; RR, reversal ratio (ratio between the IC_50_ values of CRY alone and CRY-loaded SPC liposomes); ne, not evaluable as a lower than 80% inhibition of cell viability was reached.

## References

[1] Di Sotto A., Evandri M.G., Mazzanti G. (2008). Antimutagenic and mutagenic activities of some terpenes in the bacterial reverse mutation assay. Mutat. Res..

[2] Fidyt K., Fiedorowicz A., Strządała L., Szumny A. (2016). β-caryophyllene and β-caryophyllene oxide-natural compounds of anticancer and analgesic properties. Cancer Med..

[3] Gertsch J. (2008). Antiinflammatory cannabinoids in diet—Towards a better understanding of CB2 receptor action?. Commun. Integr. Biol..

[4] Bento A.F., Marcon R., Dutra R.C., Claudino R.F., Cola M., Leite D.F., Calixto J.B. (2011). β-Caryophyllene inhibits dextran sulfate sodium-induced colitis in mice through CB2 receptor activation and PPARγ pathway. Am. J. Pathol..

[5] Chang H.J., Kim J.M., Lee J.C., Kim W.K., Chun H.S. (2013). Protective effect of betacaryophyllene; a natural bicyclic sesquiterpene; against cerebral ischemic injury. J. Med. Food.

[6] Liu H., Song Z., Liao D., Zhang T., Liu F., Zhuang K., Luo K., Yang L. (2015). Neuroprotective effects of trans-caryophyllene against kainic acid induced seizure activity and oxidative stress in mice. Neurochem. Res..

[7] Viveros-Paredes J.M., González-Castañeda R.E., Gertsch J., Chaparro-Huerta V., López-Roa R.I., Vázquez-Valls E., Beas-Zarate C., Camins-Espuny A., Flores-Soto M.E. (2017). Neuroprotective Effects of β-Caryophyllene against Dopaminergic Neuron Injury in a Murine Model of Parkinson’s Disease Induced by MPTP. Pharmaceuticals.

[8] Di Sotto A., Mazzanti G., Carbone F., Hrelia P., Maffei F., Mazzanti G. (2010). Inhibition by beta-caryophyllene of ethyl methanesulfonate-induced clastogenicity in cultured human lymphocytes. Mutat. Res..

[9] Di Giacomo S., Mazzanti G., Di Sotto A. (2016). Mutagenicity of cigarette butt waste in the bacterial reverse mutation assay: The protective effects of β-caryophyllene and β-caryophyllene oxide. Environ. Toxicol..

[10] Di Giacomo S., Abete L., Cocchiola R., Mazzanti G., Eufemi M., Di Sotto A. (2018). Caryophyllane sesquiterpenes inhibit DNA-damage by tobacco smoke in bacterial and mammalian cells. Food Chem. Toxicol..

[11] Hanušová V., Caltová K., Svobodová H., Ambrož M., Skarka A., Murínová N., Králová V., Tomšík P., Skálová L. (2017). The effects of β-caryophyllene oxide and trans-nerolidol on the efficacy of doxorubicin in breast cancer cells and breast tumor-bearing mice. Biomed. Pharmacother..

[12] Di Giacomo S., Di Sotto A., El-Readi M.Z., Mazzanti G., Wink M. (2017). Chemosensitizing Properties of β-Caryophyllene and β-Caryophyllene Oxide in Combination with Doxorubicin in Human Cancer Cells. Anticancer Res..

[13] Fontes L.B.A., Dias D.D.S., Aarestrup B.J.V., Aarestrup F.M., Da Silva Filho A.A., Corrêa J.O.D.A. (2017). β-Caryophyllene ameliorates the development of experimental autoimmune encephalomyelitis in C57BL/6 mice. Biomed. Pharmacother..

[14] Zhou L., Zhan M.L., Tang Y., Xiao M., Li M., Li Q.S., Yang L., Li X., Chen W.W., Wang Y.L. (2018). Effects of β-caryophyllene on arginine ADP-ribosyltransferase 1-mediated regulation of glycolysis in colorectal cancer under high-glucose conditions. Int. J. Oncol..

[15] Kawabata Y., Wada K., Nakatani M., Yamada S., Onoue S. (2011). Formulation design for poorly water-soluble drugs based on biopharmaceutics classification system: Basic approaches and practical applications. Int. J. Pharm..

[16] Sarfraz M., Afzal A., Raza S.M., Bashir S., Madni A., Khan M.W., Ma X., Xiang G. (2017). Liposomal co-delivered oleanolic acid attenuates doxorubicin-induced multi-organ toxicity in hepatocellular carcinoma. Oncotarget.

[17] Yang G., Yang T., Zhang W., Lu M., Ma X., Xiang G. (2014). In vitro and in vivo antitumor effects of folatetargeted ursolic acid stealth liposome. J. Agric. Food Chem..

[18] Coimbra M., Isacchi B., van Bloois L., Torano J.S., Ket A., Wu X., Broere F., Metselaar J.M., Rijcken C.J., Storm G. (2011). Improving solubility and chemical stability of natural compounds for medicinal use by incorporation into liposomes. Int. J. Pharm..

[19] Rodríguez J., Martín M.J., Ruiz M.A., Clares B. (2016). Current encapsulation strategies for bioactive oils: From alimentary to pharmaceuical perspectives. Food Res. Int..

[20] Mishra G.P., Bagui M., Tamboli V., Mitra A.K. (2011). Recent applications of liposomes in ophthalmic drug delivery. J. Drug Deliv..

[21] Lladó V., López D.J., Ibarguren M., Alonso M., Soriano J.B., Escribá P.V., Busquets X. (2014). Regulation of the cancer cell membrane lipid composition by NaCHOleate: Effects on cell signaling and therapeutical relevance in glioma. Biochim. Biophys. Acta.

[22] Kannan V., Balabathula P., Divi M.K., Thoma L.A., Wood G.C. (2015). Optimization of drug loading to improve physical stability of paclitaxel-loaded long-circulating liposomes. J. Liposome Res..

[23] Bangham A.D. (1978). Properties and uses of lipid vesicles: An overview. Ann. N. Y. Acad. Sci..

[24] Yoshida Y., Furuya E., Tagawa K. (1980). A direct colorimetric method for the determination of phospholipids with dithiocyanatoiron reagent. J. Biochem..

[25] Di Sotto A., Mazzanti G., Savickiene N., Staršelskyte R., Baksenskaite V., Di Giacomo S., Vitalone A. (2014). Antimutagenic and antioxidant activity of a protein fraction from aerial parts of Urtica dioica. Pharm. Biol..

[26] International Organization for Standardization (2009). Biological Evaluation of Medical Devices—Part 5: Tests for In Vitro Cytotoxicity (ISO 10993-5).

[27] Botrè F., Esposito S., de la Torre X., Schanzer W., Geyer H., Gotzmann A., Mareck U. (2011). How we risk: Liposomes and steroids. Recent Advances in Doping Analysis.

[28] Marsh D. (2010). Liquid-ordered phases induced by cholesterol: A compendium of binary phase diagrams. Biochim. Biophys. Acta.

[29] Sköld M., Karlberg A.T., Matura M., Börje A. (2006). The fragrance chemical β-caryophyllene-air oxidation and skin sensitization. Food Chem. Toxicol..

[30] Liu H., Yang G., Tang Y., Cao D., Qi T., Qi Y., Fan G. (2013). Physicochemical characterization and pharmacokinetics evaluation of β-caryophyllene/β-cyclodextrin inclusion complex. Int. J. Pharm..

[31] Lou J., Teng Z., Zhang L., Yang J., Ma L., Wang F., Tian X., An R., Yang M., Zhang Q. (2017). β-Caryophyllene/Hydroxypropyl-β-Cyclodextrin Inclusion Complex Improves Cognitive Deficits in Rats with Vascular Dementia through the Cannabinoid Receptor Type 2—Mediated Pathway. Front. Pharmacol..

[32] Quintans-Júnior L.J., Araújo A.A., Brito R.G., Santos P.L., Quintans J.S., Menezes P.P., Serafini M.R., Silva G.F., Carvalho F.M., Brogden N.K. (2016). β-caryophyllene; a dietary cannabinoid; complexed with β-cyclodextrin produced anti-hyperalgesic effect involving the inhibition of Fos expression in superficial dorsal horn. Life Sci..

[33] De Oliveira Neves J.K., Apolinário A.C., Alcantara Saraiva K.L., da Silva D.T.C., de Freitas Araújo Reis M.Y., de Lima Damasceno B.P.G., Pessoa A., Moraes Galvão M.A., Soares L.A.L., Veiga Júnior V.F. (2018). Microemulsions containing Copaifera multijuga Hayne oil-resin: Challenges to achieve an efficient system for β-caryophyllene delivery. Ind. Crops Prod..

[34] Li C., Zhang J., Zu Y.J., Nie S.F., Cao J., Wang Q., Nie S.P., Deng Z.Y., Xie M.Y., Wang S. (2015). Biocompatible and biodegradable nanoparticles for enhancement of anti-cancer activities of phytochemicals. Chin. J. Nat. Med..

[35] Akopian D., Kawashima R.L., Medh J.D. (2015). Phosphatidylcholine-Mediated Aqueous Diffusion of Cellular Cholesterol Down-Regulatesthe ABCA1 Transporter in Human Skin Fibroblasts. Int. J. Biochem. Res. Rev..

[36] Nuñez-Garcia M., Gomez-Santos B., Buqué X., García-Rodriguez J.L., Romero M.R., Marin J.J.G., Arteta B., García-Monzón C., Castaño L., Syn W.K. (2017). Osteopontin regulates the cross-talk between phosphatidylcholine and cholesterol metabolism in mouse liver. J. Lipid Res..

[37] Simons K., Ehehalt R. (2002). Cholesterol lipid rafts and disease. J. Clin. Investig..

[38] Litz J.P., Thakkar N., Portet T., Keller S.L. (2016). Depletion with Cyclodextrin Reveals Two Populations of Cholesterol in Model Lipid Membranes. Biophys. J..

[39] Sarpietro M.G., Di Sotto A., Accolla M.L., Castelli F. (2015). Differential Scanning Calorimetry Study on the Interaction of β-Caryophyllene and β-Caryophyllene Oxide with Phospholipid Bilayers. Thermochim. Acta.

